# Clinicopathological significance of microRNA‐21 in extracellular vesicles of pleural lavage fluid of lung adenocarcinoma and its functions inducing the mesothelial to mesenchymal transition

**DOI:** 10.1002/cam4.2928

**Published:** 2020-02-24

**Authors:** Shiori Watabe, Yoshinao Kikuchi, Shigeki Morita, Daisuke Komura, Satoe Numakura, Arisa Kumagai‐Togashi, Masato Watanabe, Noriyuki Matsutani, Masafumi Kawamura, Masanori Yasuda, Hiroshi Uozaki

**Affiliations:** ^1^ Department of Pathology Teikyo University School of Medicine Itabashi‐ku Tokyo Japan; ^2^ Department of Pathology Saitama Medical University International Medical Center Hidaka‐City Tokyo Japan; ^3^ Department of Pathology Mitsui Memorial Hospital Chiyoda‐ku Tokyo Japan; ^4^ Department of Preventive Medicine Graduate School of Medicine The University of Tokyo Bunkyo‐ku Tokyo Japan; ^5^ Department of Surgery Teikyo University School of Medicine Itabashi‐ku Tokyo Japan

**Keywords:** dissemination, exosome, extracellular vesicles, mesothelial to mesenchymal transition, microRNA‐21, pleural lavage fluid

## Abstract

**Background:**

Pre‐resection pleural lavage cytology is useful to predict tumor recurrence and the prognosis of lung cancer patients. Recently, extracellular vesicles (EVs) isolated from effusion specimens have come under the spotlight, and several studies showed that microRNA in EVs is associated with prognosis. MicroRNA‐21 (miR‐21) is a representative onco‐microRNA, and miR‐21 in EVs (EV‐miR‐21) promotes cancer dissemination by inducing mesothelial to mesenchymal transition (MMT) in the peritoneal cavity. In this study, we isolated EVs from pleural lavage fluid and focused on EV‐miR‐21 as a diagnostic factor with a relationship to pleural dissemination.

**Methods:**

The Cancer Genome Atlas dataset comprising of 448 cases of lung adenocarcinoma, tissue microarray of 144 cases of lung adenocarcinoma, and pleural lavage fluid of 41 cases was used to examine miR‐21 expression levels. The function of EV‐miR‐21 was investigated in vitro.

**Results:**

The miR‐21 expression level in primary sites was associated with a poor prognosis and correlated with pleural invasion of adenocarcinoma. EV‐miR‐21 levels in pleural lavage fluid were associated with positive cytology and pleural invasion in the primary sites, even in cytology‐negative cases. In vitro studies demonstrated that EV‐miR‐21 induces the MMT. Mesothelial cells in the MMT showed functions similar to cancer‐associated fibroblasts, which are an important stromal component in primary sites and disseminated pleural lesions.

**Conclusions:**

EV‐miR‐21 in pleural lavage fluid is important as a diagnostic and prognostic factor. Moreover, EV‐miR‐21 induces the MMT, which can form premetastatic niches of dissemination in the pleural cavity.

## Graphical abstract

This study demonstrates that microRNA‐21 in extracellular vesicles (EV‐miR‐21) in the pleural lavage is associated with clinicopathological parameters and with functions inducing the mesothelial to mesenchymal transition in the pleura. Factors in the EVs, such as miR‐21, could provide important cytopathological and histopathological information in the clinical laboratory setting. 

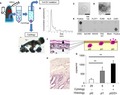

## 1 INTRODUCTION

The cancer microenvironment is important not only in cancer progression in the primary sites, but also in metastatic lesions. A metastatic niche is the cancer microenvironment that helps cancer cells progress in distant organs. Recently, a premetastatic niche was shown in hematogenous and lymphatic metastatic pathway. This theory hypothesized that cancer cells construct a microenvironment suitable for metastasis in distant organs before the metastatic phase. However, both metastatic and premetastatic niches in the dissemination pathway are not fully understood. In peritoneal dissemination, it has been reported that cancer‐associated fibroblasts (CAFs), among the representative components of the cancer microenvironment, were derived from mesothelial cells via the mesothelial to mesenchymal transition (MMT).^1^, ^2^, ^3^, ^4^ On the other hand, mechanisms of pleural dissemination remain unclear. Although the MMT of pleural mesothelial cells was also reported in pulmonary fibrosis,^5^, ^6^ there has been no report about the MMT in pleural dissemination.

Recently, exosomes have attracted attention as an intermediary to construct premetastatic niches. Exosomes are extracellular vesicles (EVs) with diameters of 30‐150 nm and round or cup‐shaped morphology, a lipid composition, and a double lipid layer.^7^ Exosomes contain many functional factors, such as proteins, lipids, microRNA, mRNA, and DNA, which enable target cells to change gene expression.^8^, ^9^, ^10^ Exosomes in peritoneal malignant ascites and peritoneal lavage in gastric cancer or ovarian cancer have recently been studied, and they have been found to promote peritoneal dissemination.^2^, ^11^, ^12^

Positive pre‐resection pleural lavage cytology is associated with local and distant tumor recurrence and unfavorable patient survival outcomes.^13^, ^14^ Therefore, cytopathological diagnosis is considered useful to predict lung cancer patients’ prognosis. We have tried to isolate EVs including exosomes from pleural lavage fluid to identify prognostic factors other than positive pleural lavage cytopathology. To the best of our knowledge, there have been no reports of extracting EVs from pre‐resection pleural lavage fluid and examining the significance of factors in EVs.

In this study, the clinicopathological significance of microRNA‐21 (miR‐21) expression levels in primary lesions of lung adenocarcinoma and in EVs extracted from pleural lavage fluid was assessed, because a growing number of studies suggest that microRNAs are important in various steps of lung carcinogenesis,^15^ and miR‐21 is expressed in many malignant tumors, including breast, stomach, prostate, colon, brain, head and neck, esophagus, pancreas, and lung cancers.^16^, ^17^ The function of miR‐21 in EVs (EV‐miR‐21) associated with pleural metastasis was also studied, focusing on the MMT in the pleural cavity.

## 2 MATERIALS AND METHODS

### 2.1 The Cancer Genome Atlas (TCGA) dataset analysis

Survival analysis: The relationship between miR‐21 expression levels and survival time was analyzed in 448 lung adenocarcinoma cases from The Cancer Genome Atlas in which both were available. First, the R‐language RTCGAToolbox^18^ was used to download the prognosis and microRNA expression levels of lung adenocarcinoma cases. Next, the 448 cases were divided into two groups, with miR‐21 expression levels that were higher (222 cases) or lower (226 cases) than the median, and Kaplan‐Meier curves were drawn using the ggsurvplot function of the survminer R package.

Gene‐set enrichment analysis (GSEA): GSEA^19^ was performed to identify gene sets that were altered between miR‐21 high and low cases. First, HTSeq‐count data from RNA‐seq of 513 lung adenocarcinoma cases in TCGA were downloaded from the GDC data portal, and 445 cases with miRNA expression information were used for the analysis. Then, the genes were preranked according to the log fold‐change shrinkage values calculated by the DESeq2 R package.^20^ The GSEAPreranked function in the javaGSEA desktop application was used to calculate normalized enrichment scores (NES) and (FDR) values for 50 Hallmark gene sets in The Molecular Signature Database (MSigDB).

### 2.2 Human samples

A total of 144 primary lung adenocarcinoma cases for the tissue microarray (TMA) study, 15 pleural dissemination nodules of seven cases, and pre‐resection pleural lavage fluids of 41 cases, were obtained from the archives of the Department of Pathology, Teikyo University Hospital, from 2000 to 2013, 2000 to 2017, and 2016 to 2017, respectively. TMAs were constructed with formalin‐fixed, paraffin‐embedded (FFPE) tissue blocks, as described previously.^21^, ^22^ These studies were approved by the ethics committee of Teikyo University School of Medicine (No. 18‐063‐2, 16 October 2018; No. 18‐041‐2, 16 October 2018).

### 2.3 Cell culture

The mouse fibroblast cell line NIH3T3 (American Type Culture Collection) was grown in Dulbecco's modified Eagle's medium (DMEM, Sigma‐Aldrich) with 10% fetal bovine serum (FBS, Sigma‐Aldrich) and 100 U/mL penicillin‐100 µg/mL streptomycin‐0.25 µg/mL amphotericin B at 37°C in a humid atmosphere saturated with 5% CO_2_. Advanced DMEM (Thermo Fisher Scientific) was used, for isolation of EVs from cultured NIH3T3. The human mesothelial cell line Met‐5A (American Type Culture Collection) was grown in Medium199 with the same supplementations and conditions. Primary cultured human lymphatic endothelial cells (HLECs) were purchased from ScienCell Research Laboratories and grown in endothelial cell medium (ScienCell Research Laboratories) according to the manufacturer's instructions.

### 2.4 Immunohistochemistry

The immunohistochemical studies were performed using primary antibodies against desmin (1:50, D33, DAKO‐Agilent), alpha smooth muscle actin (αSMA) (1:100, 1A4, DAKO‐Agilent), and calretinin (1:50, DAK Calret1, DAKO‐Agilent).

FFPE sections were stained immunohistochemically as described previously.^23^ For the study of double‐staining immunohistochemistry, the combinations were desmin and αSMA, or calretinin and αSMA. Antigen retrieval was performed by boiling at 98°C for 20 minutes in retrieval buffer (pH 6.0 for desmin and αSMA; pH 9.0 for calretinin). The expression of desmin or calretinin was first detected by visualization using the horseradish peroxidase (HRP)‐labeled polymer method (Envision FLEX system, DAKO‐Agilent), and subsequently, αSMA expression was detected on the same section using the alkaline phosphatase‐labeled polymer method (Envision G|2 system/AP, DAKO‐Agilent).

### 2.5 Immunofluorescence of cultured cells

The 24 × 24 mm^2^ cover glass on which cells were cultured was fixed with 100% methanol at −20°C. The glass was incubated in 3% goat serum (DAKO‐Agilent)/phosphate buffered saline with tween 20 (PBST) for 30 minutes. The primary antibodies against E‐cadherin (1:25, 36B5, Novocastra‐Leica), vimentin (1:200, Vim3B4, DAKO‐Agilent), and αSMA were applied overnight at 4°C. As the secondary antibody, rabbit polyclonal anti‐mouse immunoglobulins/FITC antibody (DAKO‐Agilent) was used. Nuclei were stained with 4',6‐diamidino‐2‐phenylindole (DAPI) (Prolong Diamond Antifade Mountant with DAPI, Thermo Fisher Scientific). Signal intensity of each cells was measured using WinRoof ver.5.02 (Mitani Corporation).

### 2.6 In situ hybridization (ISH) for miR‐21

TMA slides were stained with locked nucleic acid‐modified digoxigenin (DIG)‐labeled probes for miR‐21 (Exiqon), as described previously.^21^, ^22^ Each TMA core was scored independently by two pathologists, without knowledge of clinical data, for intensity of staining in cancer cells and stromal cells using the histoscore.^21^, ^22^, ^24^

### 2.7 Isolation of EVs and RNA in EVs

EVs were isolated from 1 mL of supernatant of centrifuged pre‐resection pleural lavage fluid using miRCURY Exosome Isolation Kit‐Cells, urine, and CSF (Exiqon) according to the manufacturer's instructions. This kit is a viable alternative to ultracentrifugation, even with limited biological samples.^25^ Representative isolated EV samples were verified using nanoparticle tracking analysis by Theoria Science. Positive and negative surface protein markers of exosomes were confirmed using the Exo‐Check Exosome Antibody Arrays (System Biosciences) according to the manufacturer's instructions.

Total RNA was isolated from 100‐µL EV samples using the miRCURY RNA Isolation Kit‐Cell&Plant (Exiqon), following the manufacturer's instructions. Reverse transcription reaction was performed with 5 µL of total RNA using the TaqMan MicroRNA Reverse Transcription Kit and TaqMan MicroRNA Assays RT primer (Thermo Fisher Scientific).

### 2.8 Transmission electron microscopy (TEM)

Isolated EVs were fixed in 2% paraformaldehyde. The fixed sample was absorbed to formvar‐coated copper grids for 20 minutes. The grids were fixed in 1% glutaraldehyde for 5 minutes. After being washed with distilled water (DW), the grids were stained with 2% uranyl acetate for 2 minutes. For immunoelectron microscopy, the fixed sample was absorbed to formvar‐coated nickel grids for 30 minutes. The grids were incubated with primary antibody for CD63 (8A12, Cosmo Bio) in PBS for 2 hours at room temperature. After being washed with PBS and DW, the grids were subsequently incubated with secondary antibody conjugated to 10‐nm gold particles (goat anti‐mouse IgG polyclonal antibody, BBI solutions) for 90 minutes at room temperature and washed with DW. The grids were fixed in 1% glutaraldehyde for 5 minutes. After being washed with DW, the grids were stained with 2% uranyl acetate for 2 minutes. TEM was used to observe EV morphology (Hitachi H7650 microscope, Hitachi High‐Technologies Corporation).

### 2.9 Digital polymerase chain reaction (PCR)

TaqMan MicroRNA Assays (hsa‐miR‐21, Applied Biosystems‐Thermo Fisher Scientific) were used to examine the expression of miR‐21 in EVs isolated from 1 mL of pleural lavage fluid. Copy numbers of EV‐miR‐21 in EVs were measured with cDNA samples diluted 5× using the QuantStudio 3D Digital PCR system (Thermo Fisher Scientific) and analyzed using the QuantStudio 3D AnalysisSuite Cloud Software (Thermo Fisher Scientific) according to the manufacturer's instructions.

### 2.10 Transduction of miR‐21 mimic and precursor‐miR‐21 (pre‐miR‐21)

Lentivirus transduction of miR‐21 mimic and control for NIH3T3 was performed using XMIRXpress lentivector miRNA‐21‐5p with Xmotif or XMIRXpress lentivector Nontargeting miRNA with Xmotif (System Biosciences) using the kit, LentiX Packaging Shingle Shots (Clontech‐Laboratories‐Takara Bio), according to the manufacturer's instructions. Met‐5A was transduced to pre‐miR‐21 using hsa‐mir‐21 Lentiviral stock or hsa‐mir‐ctrl Lentiviral stock (Biosettia).

### 2.11 Coculture

Separate co‐cultures were performed using 6‐well plates and 0.4‐µm pore size inserts, with each cell in the wells or inserts seeded (10^5^ cells) for 3 days.

### 2.12 Wound healing assay

The wound healing assay was performed with nearly confluent cultured cells in the 6‐well plate, as described previously.^10^ To quantify cell migration, the wound width of three different points was measured after 0, 8, 16, and 24 hours by normalization with the initial wound width.

### 2.13 Type‐1 collagen gel contraction assay

A contraction assay using type‐1 collagen gel was performed, as described previously.^26^ Preparation of collagen gel matrix was performed using Cellmatrix (type1‐A, Nitta Gelatin, Osaka, Japan) according to the manufacturer's instructions. Briefly, cultured cells (5 × 10^5^ cells) were suspended in 800 µL of Cellmatrix Type1‐A. Collagen gel matrix was added to each well preconditioned with FBS and incubated for 5 minutes at 37°C in a humid atmosphere saturated with 5% CO_2_. After collagen gel matrix polymerization, 3 mL of complete DMEM was added. To initiate collagen gel contraction after 24 hours, polymerized gels were gently released from the underlying culture dish. The degree of collagen gel contraction was determined after 2, 4, 6, and 10 days by measuring the contraction percentage ([area of the well bottom—area of the gel/area of the well bottom] ×100).

### 2.14 Statistical analysis

The relationships between miR‐21 expression and patients’ clinicopathological features were evaluated using the Chi‐squared test. Survival was analyzed using the Kaplan‐Meier method, with curves compared using the log‐rank test. All other numerical results are expressed as means ± SEM. Data were evaluated using the Mann‐Whitney *U* test. *P* < .05 was considered significant. Statistical analyses were performed using JMP Pro (SAS institute).

## 3 RESULTS

### 3.1 Clinicopathological significance of miR‐21 expression in primary lung adenocarcinomas

Whether the miR‐21 expression level could be a prognostic factor was first examined using publicly available data from TCGA including 448 cases of lung adenocarcinoma. This in silico study demonstrated a significant association between a high miR‐21 expression level and a poor prognosis (*P* = .0051, Figure S1A). To clarify the localization of miR‐21 expression in lung adenocarcinomas in detail, miR‐21 expression was then carefully evaluated in cancer cells and in stromal cells by ISH using TMA including 144 cases of lung adenocarcinoma. The patients’ median age was 71 (interquartile range, 42‐87) years, and the group consisted of 80 males and 64 females. The miR‐21 expression level was evaluated using a semi‐quantitative scoring system, as reported previously (Figure S1B).^21^, ^22^, ^24^ Ninety‐six cases (66.7%) showed strong miR‐21 positivity (histoscores 2 and 3) in cancer cells, and 82 cases (56.9%) showed strong miR‐21 positivity in the stromal cells. The levels of miR‐21 positivity were examined in association with clinicopathological features (Table 1). High expressions of miR‐21 in cancer cells and stromal cells each showed significant associations with pleural invasion (cancer cells, *P* = .012; stromal cells, *P* = .0037), although both were not related to T stage. In addition, high expression in stromal cells was also associated with lymph node metastasis and vascular invasion (*P* = .0104 and *P* = .0055). Furthermore, high miR‐21 expression in cancer cells and stromal cells was related to poor prognosis on survival analysis (Figure S1C, D). High miR‐21 positivity in cancer cells showed worse disease‐free survival (*P* = .0212). High miR‐21 positivity in stromal cells showed worse disease‐free survival and overall survival (*P* = .0007 and *P* = .0093). These data demonstrated that miR‐21 is a useful prognostic biomarker in primary lung adenocarcinomas.

**Table 1:** Clinicopathological features and miR‐21 positivity of cancer and stromal cells

Clinicopathological features	n	Cancer cells miR‐21			Stromal cells miR‐21		
		Low	High	*P* value	Low	High	*P* value
Age (y)							
≦60	20	6	14		8	12	
＞60	124	42	82	.7333	54	70	.7662
Gender							
Male	80	26	54		32	48	
Female	64	22	42	.8125	30	34	.4077
T stage							
T1	78	28	50		36	42	
T2‐3	62	19	43	.5133	26	36	.6177
Lymph node metastasis							
Negative	119	42	77		57	62	
Positive	25	6	19	.2762	5	20	**.0104**
Pleural invasion							
Negative	90	37	53		49	41	
Positive	46	9	37	**.0120**	13	33	**.0037**
Vascular invasion							
Negative	109	37	72		54	55	
Positive	32	9	23	.5370	7	25	**.0055**
Lymphatic permeation							
Negative	109	36	73		50	59	
Positive	32	10	22	.8504	11	21	.2484

Bolded values are statistically significant at *P* < .05.

### 3.2 EV‐miR‐21 in pre‐resection pleural lavage fluid associated with its cytopathological results and pleural invasion in primary sites

**Figure 1:**
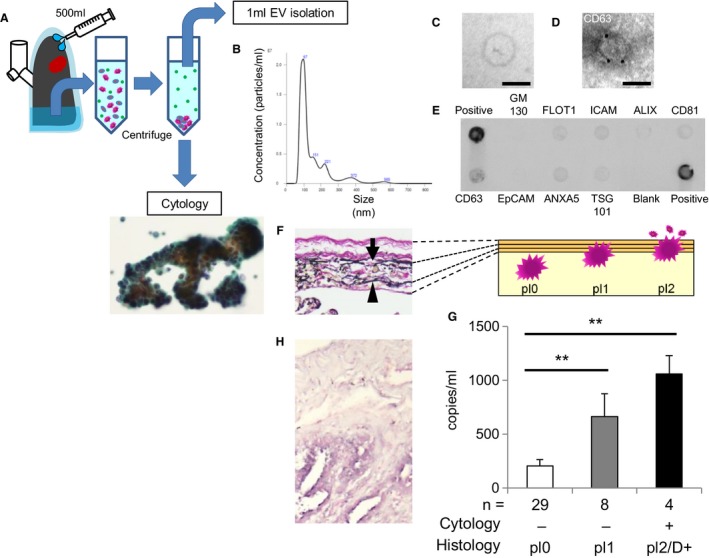
Clinicopathological significance of microRNA‐21 expression in extracellular vesicles of pleural lavage fluid. A, Schematic representation of pleural lavage cytology and isolation of extracellular vesicles (EVs). Cytologic image of cancer cells in pleural lavage fluid (Cytology). B, Representative image of the nanoparticle tracking analysis for isolated EVs. Isolated EVs are also confirmed using C, transmission electron microscopy (bar, 50 nm), D, immunoelectron microscopy for CD63 (bar, 50 nm), and E, positive (FLOT1, ICAM, ALIX, CD81, CD63, EpCAM, ANXA5, TSG101) and negative (GM130) surface protein markers of exosome. F, Histologic image of pleura (Elastica van Gieson stain; arrow, external elastic membrane; arrowhead, internal elastic membrane) (left). Schematic representation for pl0 (nonpleural invasion), pl1 (pleural invasion without pleural exposure), and pl2 (pleural exposure) (right). (G) Copy numbers of microRNA‐21 (miR‐21) in EVs of pleural lavage fluid in the cytology‐positive pl2 (pleural exposure)/D+ (clinically pleural dissemination) cases (black), cytology‐negative pl1 cases (gray), and cytology‐negative pl0 cases (white) (**P < .01). H, Histologic image of in situ hybridization for miR‐21 in the region of pleural invasion

With the hypothesis that there are other prognostic biomarkers in the supernatant of centrifuged pleural lavage fluid after precipitating cells for cytopathology, the focus was on EVs including microRNA. EVs were isolated from pre‐resection pleural lavage fluid, and pleural lavage EVs were detected using nanoparticle tracking analyses to assess size distribution (Figure 1A,B). EVs with a peak in the 70‐130 nm‐diameter range were observed. Transmission electron microscopy revealed that isolated EVs showed typical size and morphology (Figure 1C). The isolated EVs were also analyzed detecting the conventional exosome membrane markers including CD63 by immunoelectron microscopy and antibody arrays (Figure 1D,E).

Then, whether the EV‐miR‐21 expression level in pleural lavage fluid was associated with cancer cells in the pleural cavity was investigated. The EV‐miR‐21 copy number was measured using digital PCR in 41 cases that were also studied with cytopathology and histopathologic diagnosis of the primary lesions. Pleural invasion (pl1) was diagnosed when cancer cells invaded the external elastic membrane (Figure 1F). In this study, cytopathology‐positive cases of pleural lavage fluid (Figure 1A) demonstrated pleural exposure (pl2) or clinically pleural dissemination (D+). Interestingly, positive pleural lavage fluid showed high copy numbers compared to negative pleural lavage fluid without pleural invasion in primary sites (*P* < .01, Figure 1G). On ISH studies for the primary lesions of cases with positive pleural lavage cytopathology, miR‐21 signals were observed at the site of pleural invagination (Figure 1H). Furthermore, even in cases with negative pleural lavage cytopathology, cases with pleural invasion (without pleural exposure) showed significantly higher copy numbers than cases without pleural invasion (*P* < .01, Figure 1G). These data demonstrated that the pleural lavage fluid EV‐miR‐21 expression level reflected the presence of cancer cells in the pleural cavity, and the EV‐miR‐21 expression level in the pleural cavity was increased at the stage of pleural invasion even before pleural exposure.

### 3.3 Function of miR‐21 in lung adenocarcinomas studied by GSEA using TCGA data

To investigate the function of miR‐21 in lung adenocarcinomas, pathway analysis was performed using GSEA comparing the high and low miR‐21 expression cases of TCGA. It showed that the up‐regulated gene sets in high expression cases were related to apoptosis and the epithelial‐mesenchymal transition (EMT). They were also associated with TNFα signaling via NFKβ, IL6‐JAK‐STAT3 signaling, and interferon *α*, interferon *γ*, and inflammatory responses (Figure S2). It is supposed that the functions of miR‐21 for apoptosis avoidance and the EMT are advantageous for cancer cells surviving in the pleural cavity, because they have to proliferate in an anchorage‐independent manner. Therefore, a high EV‐miR‐21 expression level in the pleural cavity possibly promotes cancer cells and dissemination. EV‐miR‐21 actions on mesothelial cells in the pleural cavity were also examined because mesothelial cells are known to be important components in the microenvironment of cancer dissemination. It has been reported that the MMT is caused in the same manner as the EMT.^1^

### 3.4 CAFs derived from mesothelial cells in the pleural dissemination of lung adenocarcinomas

**Figure 2:**
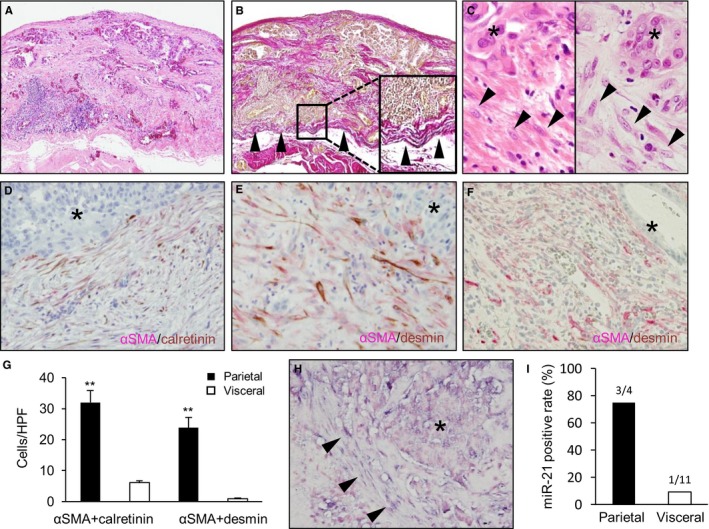
Mesothelial to mesenchymal transition in pleural dissemination of lung adenocarcinoma. A, B, Representative histological images of a pleural disseminated lesion (A, H&E stain; B, Elastica van Gieson stain). B, Disseminated tumor is mainly observed in the outside of the external elastic membrane (arrowhead). C, Cancer‐associated fibroblasts (CAFs) in the stroma of visceral (left) and parietal (right) disseminated lesions (*, tumor; arrowhead, CAFs). D, E, CAFs double positive for αSMA and calretinin (D; *, tumor), or αSMA and desmin (E; *, tumor) in pleural dissemination. F, CAFs positive for αSMA but negative for desmin in a primary lesion (*, tumor). G, The numbers of CAFs double positive for αSMA and calretinin or αSMA and desmin in the parietal and visceral disseminated lesions (**P < .01). H, microRNA‐21 (miR‐21) expression of CAFs in a pleural disseminated lesion (*, tumor; arrowhead, CAFs). I, Positive rates of nodules showing stromal miR‐21 expression (in situ hybridization histoscores 2 and 3) in the parietal and visceral lesions (3 of 4 parietal nodules; 1 of 11 visceral nodules)

In peritoneal dissemination, the MMT is important in the cancer microenvironment.^1^, ^2^, ^3^ To assess whether the MMT is also observed in the cancer microenvironment in pleural dissemination, surgically resected pleural disseminated nodules of lung adenocarcinomas were studied (Figure 2A‐C) by double‐staining immunohistochemistry using calretinin, desmin, and αSMA. Mesothelial cells are usually immunoreactive for calretinin, and activated mesothelial cells also show desmin positivity.^27^ However, neither nonactivated nor activated fibroblasts such as CAFs show immunoreactivity to them. In addition, αSMA is a marker of activated fibroblasts, and mesothelial cells and nonactivated fibroblasts are negative for αSMA (Table S1).^28^, ^29^, ^30^, ^31^ In the studies of pleural disseminated nodules, double‐positive staining for αSMA and calretinin or αSMA and desmin was observed in stromal cells (Figure 2D,E). On the other hand, double‐staining immunohistochemistry at the primary site of pleural invasion demonstrated only αSMA positivity (Figure 2F). TMA was also examined, showing no significant immunoreactivity for desmin in stromal cells in the primary lesions. Stromal cells in the disseminated nodules were considered CAFs derived from mesothelial cells, because of double‐positivity for both CAF and mesothelial cell markers. This indicated that the MMT was also observed in the microenvironment of pleural dissemination, similar to that seen in abdominal dissemination. However, there were differences between parietal and visceral dissemination. The numbers of both types of double‐positive cells were higher in parietal disseminated lesions than in visceral disseminated lesions (Figure 2G). Furthermore, they also showed strong miR‐21 expression by ISH (Figure 2H), and the rate of disseminated nodules positive for stromal miR‐21 ISH (histoscores 2 and 3) was significantly elevated in parietal lesions (Figure 2I). These histochemical studies and GSEA results indicate that the MMT in pleural dissemination is associated with miR‐21.

### 3.5 MMT and its function equivalent to CAFs induced by EV‐miR‐21

To investigate the detailed function of EV‐miR‐21 related to mesothelial cells, NIH3T3 overexpressing miR‐21 mimic in EVs (NIH3T3‐miR‐21‐mimic) was constructed using commercially available lentivirus vectors, including arrangement of an miR‐21 mimic with a special tag that tends to be encapsulated in EVs.

**Figure 3:**
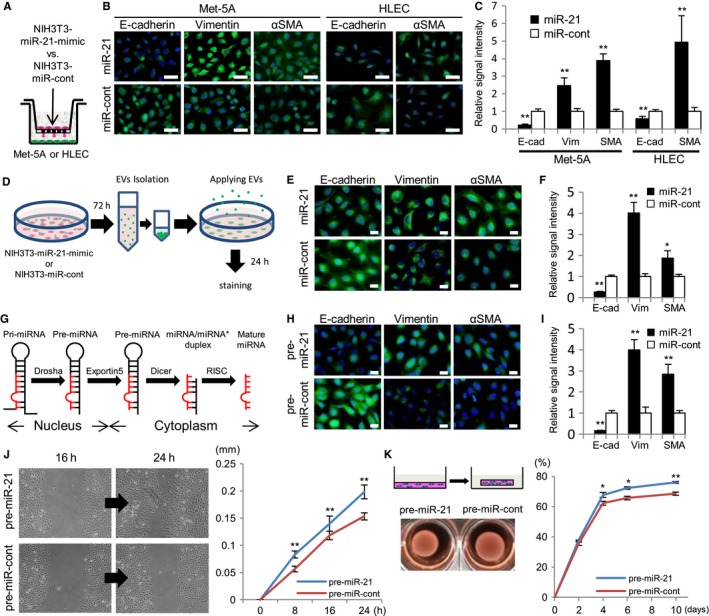
Mesothelial to mesenchymal transition and its function equivalent to cancer‐ associated fibroblasts induced by microRNA‐21 in extracellular vesicles: A, Separate cocultured studies of mesothelial cells (Met‐5A) or lymphatic endothelial cells (HLECs) with mouse fibroblast cell line (NIH3T3) producing microRNA‐21 mimic in extracellular vesicles (NIH3T3‐miR‐21‐mimic) and NIH3T3‐miR‐control. B, Met‐5A cocultured with NIH3T3‐miR‐21‐mimic demonstrates decreased E‐cadherin and increased vimentin and αSMA immunoreactivity. HLECs cocultured with NIH3T3‐miR‐21‐mimic also demonstrate decreased E‐cadherin and increased αSMA immunoreactivity (bar, 50 µm). C, Fluorescence signal intensities of each cell are also measured and they show statistically significant differences (E‐cad, E‐cadherin; Vim, vimentin; SMA, αSMA). D, Schematic representation of protocol for isolated extracellular vesicles (EVs) from NIH3T3‐miR‐21‐mimic or NIH3T3‐miR‐control directly applying to Met‐5A. E, F, Met‐5A incubated with EVs from NIH3T3‐miR‐21‐mimic showing decreased E‐cadherin and increased vimentin and αSMA immunoreactivity (E bar, 20 µm; F *, *P *< .05; **, *P *< .01). G, Nuclear and cytoplasmic processing of microRNA (miRNA). The pre‐miRNA is cleaved by the RNase III enzyme (Dicer), incorporated into the RNA‐induced silencing complex (RISC), and becomes mature miRNA in the cytoplasm. H, I, Met‐5A overexpressing pre‐miR‐21 showing decreased E‐cadherin and increased vimentin and αSMA immunoreactivity (H bar, 20 µm; I **P < .01). J, Wound healing assay and (K) collagen gel contraction assay of Met‐5A overexpressing pre‐miR‐21 and control (*P < .05; **P < .01)

It has been reported that mesothelial cells showed increased vimentin and αSMA expressions and decreased E‐cadherin expression during the MMT process.^1^, ^2^, ^3^, ^32^, ^33^, ^34^ Therefore, immunoreactivity of a human mesothelial cell line (Met‐5A) separately cocultured with NIH3T3‐miR‐21‐mimic (Figure 3A) was studied. In this condition, EVs derived from NIH3T3 act on mesothelial cells. It was found that Met‐5A cocultured with the NIH3T3‐miR‐21‐mimic showed increased immunoreactivity to vimentin and αSMA and decreased immunoreactivity to E‐cadherin compared to Met‐5A cocultured with control NIH3T3‐miR‐control (Figure 3B). Human lymphatic endothelial cells (HLECs), which have common immunohistochemical markers with mesothelial cells, such as D2‐40, also showed increased αSMA and decreased E‐cadherin expressions in the same cocultured condition (Figure 3B). No difference was observed in vimentin, because lymphatic endothelial cells intrinsically showed strong vimentin expression. Fluorescence signal intensities of each cell were also measured and they showed statistically significant differences (Figure 3C). Then, we isolated EVs from each cultured medium of NIH3T3‐miR‐21‐mimic and NIH3T3‐miR‐control, and applied them to Met‐5A (Figure 3D). Met‐5A incubated with EVs from NIH3T3‐miR‐21‐mimic also showed increased vimentin and αSMA, and decreased E‐cadherin expression (Figure 3E,F). These data demonstrated that EVs rich in miR‐21 induced mesenchymal transitions of mesothelial and lymphatic endothelial cells.

Furthermore, pre‐miR‐21 overexpressing Met‐5A (Met‐5A‐pre‐miR‐21) was constructed (Figure 3G). These cells had downregulated expression of PDCD4, a target gene of mature miR‐21. Met‐5A‐pre‐miR‐21 showed increased immunoreactivity to vimentin and αSMA and decreased immunoreactivity to E‐cadherin (Figure 3H,I). Whether mesothelial cells overexpressing miR‐21 gained the function of CAFs was also examined. CAFs have similar functions to myofibroblasts (activated fibroblasts), and they show more migratory and contractile activities than normal nonactivated fibroblasts.^29^ Therefore, the migratory and contractile activities of Met‐5A‐pre‐miR‐21 were studied. The wound healing assay demonstrated that Met‐5A‐pre‐miR‐21 showed earlier wound closure than control cells (Figure 3J). The contraction assay using the type‐1 collagen gel also demonstrated high contractile activity of Met‐5A‐pre‐miR‐21 compared to control cells (Figure 3K). Migratory and contractile activities are known to be important functions of CAFs in the cancer microenvironment. These data indicated that miR‐21 induced the MMT of mesothelial cells, and they changed into fibroblastic cells as CAFs in pleural dissemination.

## 4 DISCUSSION

Cytopathological positivity for cancer cells in pleural lavage fluid is considered a precursor condition of pleural dissemination or a malignant pleural effusion.^13^, ^14^ In this study, EVs including exosomes were isolated in pleural lavage fluid. This is the first report to examine EVs from pleural lavage fluid, although there are some studies isolating EVs from peritoneal malignant ascites or peritoneal lavage fluid in abdominal organ cancers such as gastric cancer.^2^, ^11^, ^12^ These previous studies demonstrated that EVs associated with cancer cells in the peritoneal cavity can be prognostic factors, and they function to promote peritoneal dissemination.^2^, ^3^, ^4^, ^11^, ^12^ The present studies demonstrated that the EV‐miR‐21 expression level in pleural lavage fluid reflected not only cytopathological positivity for cancer cells in pleural lavage fluid, but also the degree of pleural invasion at the primary sites. As in our previous studies of gastric and bladder cancers,^21^, ^22^ miR‐21 expression levels in cancer cells and stromal cells of lung adenocarcinomas in primary sites were also prognostic. Taken together, miR‐21 expressions in the primary lesion and in the pleural cavity of lung adenocarcinoma cases can be important diagnostic and prognostic factors.

The mechanism of dissemination remains unclear. Recently, pre‐metastatic niche formation has been considered an important mechanism in hematogenous and lymphatic metastases. Furthermore, mechanisms of organ tropic metastasis, first mentioned by Stephen Paget as the “Seed and soil hypothesis,” are partially explained by cancer‐derived EVs (exosomes) that induce premetastatic niches in targeted organs.^9^, ^35^ The existence of premetastatic niches in the dissemination pathway is uncertain, but Lee et al recently demonstrated that a premetastatic niche is formed in the omentum in ovarian cancer, showing that neutrophilic infiltration and extracellular trap formation in the omental milky spots are important in the formation of omental dissemination.^36^

Functions of mesothelial cells are also an area of focus in studying the mechanisms of peritoneal dissemination. There are two major hypotheses associated with the role of mesothelial cells in peritoneal dissemination. One is that mesothelial cells have protective roles in the peritoneal cavity, EVs derived from cancer cells cause apoptosis of mesothelial cells, and cancer cells disseminate to the foci of the destroyed mesothelial layer.^4^, ^11^ The other is that mesothelial cells promote dissemination by the MMT, which causes mesothelial cells to change into cancer stromal cells such as CAFs.^1^, ^2^, ^3^ EV‐miR‐21 in the peritoneal cavity was recently demonstrated to induce the MMT in gastric cancer.^3^ Current studies also showed that the MMT was induced in pleural dissemination, and that it can be caused by EV‐miR‐21. The in vitro study demonstrated that mesothelial cells showing the MMT acquired functions similar to those of CAFs, such as migration and contractile activity in collagen gel. These data indicate that the MMT is an important mechanism in both peritoneal and pleural dissemination.

The pleural lavage study also demonstrated that the EV‐miR‐21 expression level in the pleural cavity was upregulated before pleural exposure of cancer cells. EV‐miR‐21 upregulation at the pre‐dissemination stage may promote cancer cell survival in the pleural cavity, since miR‐21 can prevent cancer cell apoptosis as shown in the present GSEA and demonstrated in a previous study.^10^ Furthermore, it may construct a premetastatic niche by inducing the MMT in the pleural cavity.

The mechanism of EV‐miR‐21 upregulation before pleural exposure of cancer cells remains unclear. However, positive pleural lavage cytopathology without pleural exposure of cancer cells in the primary lesions is infrequently seen. Liu et al demonstrated that cancer cells in the lymphatic cisterns permeate into the pleural cavity via adjacent stomata in the pleural ligament.^37^ They hypothesized that sub‐pleural lymphatic invasion might be a prerequisite for pleural spread. A similar mechanism can be considered in the EV‐miR‐21 upregulation before pleural exposure of cancer cells. EV‐miR‐21 at the site of pleural invasion permeates into the subpleural lymphatics, and they then may reach lymphatic cisterns in the pleural ligament and be released into the pleural cavity via stomata.

MMT means myofibroblastic conversion of mesothelial cells, characterized by an increase in the invasive capacity that allows them to invade the peritoneal compact zone.^1^ Sandoval et al suggested that the MMT renders the peritoneum more receptive to tumor cell attachment/invasion and contributes to secondary tumor growth by promoting its vascularization.^1^ The present study proved that the MMT is observable in pleural dissemination. In addition, the MMT was better observed in parietal dissemination than in visceral dissemination. We hypothesized it was attributed to the fact that a pleural effusion is physiologically absorbed by parietal pleura via intermesothelial stomata that connect to lymphatic vessels of the thoracic wall.^38^ Therefore, EVs in the pleural cavity can affect parietal pleura more than visceral pleura. The MMT is also known to cause ultrafiltration failure in peritoneal dialysis, and a high glucose level is a major biological mechanism leading to myofibroblast accumulation derived from mesothelial cells in the peritoneal cavity.^32^ In the pleural cavity, glucose levels may differ between parietal and visceral pleura, because the former is fed by the intercostal artery, whereas the latter is in the periphery of the lung parenchyma. In addition, intermesothelial stomata are usually observed only in the parietal pleura, except for the pleural ligament. This structural difference of the mesothelial cell layer can affect MMT incidence.

In conclusion, this study showed that pleural lavage fluid EVs potentially include important diagnostic and prognostic factors, although the numbers of cases with positive pleural lavage fluid cytopathology were limited. In addition, EV‐miR‐21 in the pleural cavity is important, not only in association with clinicopathological parameters, but also in functions inducing the MMT, which results in progressive pleural dissemination. Examining the factors such as miR‐21 in the EVs can be applicable for a clinical laboratory test and give additional information on cytopathological and histopathological diagnosis.

## CONFLICT OF INTEREST

The authors declare no conflict of interest regarding this study.

## AUTHOR CONTRIBUTIONS

Shiori Watabe: Conceptualization, data curation, formal analysis, investigation, methodology, writing–original draft, and writing–review and editing, Yoshinao Kikuchi: Conceptualization, data curation, formal analysis, investigation, methodology, supervision, funding acquisition, writing–original draft, and writing–review and editing, Shigeki Morita: Data curation, formal analysis, methodology, and writing–review and editing, Daisuke Komura: Data curation, formal analysis, methodology, and writing–review and editing, Satoe Numakura: Data curation and formal analysis, Arisa Kumagai‐Togashi: Data curation and formal analysis, Masato Watanabe: Data curation and formal analysis, Noriyuki Matsutani: Conceptualization, data curation, supervision, and writing–review and editing, Masafumi Kawamura: Conceptualization, supervision, and writing–review and editing, Masanori Yasuda: Conceptualization, supervision, and writing–review and editing, Hiroshi Uozaki: Conceptualization, supervision, and writing–review and editing.

## Data Availability

The data that support the findings of this study are available from the corresponding author upon reasonable request.
