# Supplementary material for: Clinicopathological significance of microRNA‐21 in extracellular vesicles of pleural lavage fluid of lung adenocarcinoma and its functions inducing the mesothelial to mesenchymal transition
Source: Cancer Med. 2020 Feb 24;9(8):2879–90. doi: 10.1002/cam4.2928 (PMC7163097; doi:10.1002/cam4.2928)
Supplement: Supplementary file 1 [file CAM4-9-2879-s001.pdf]

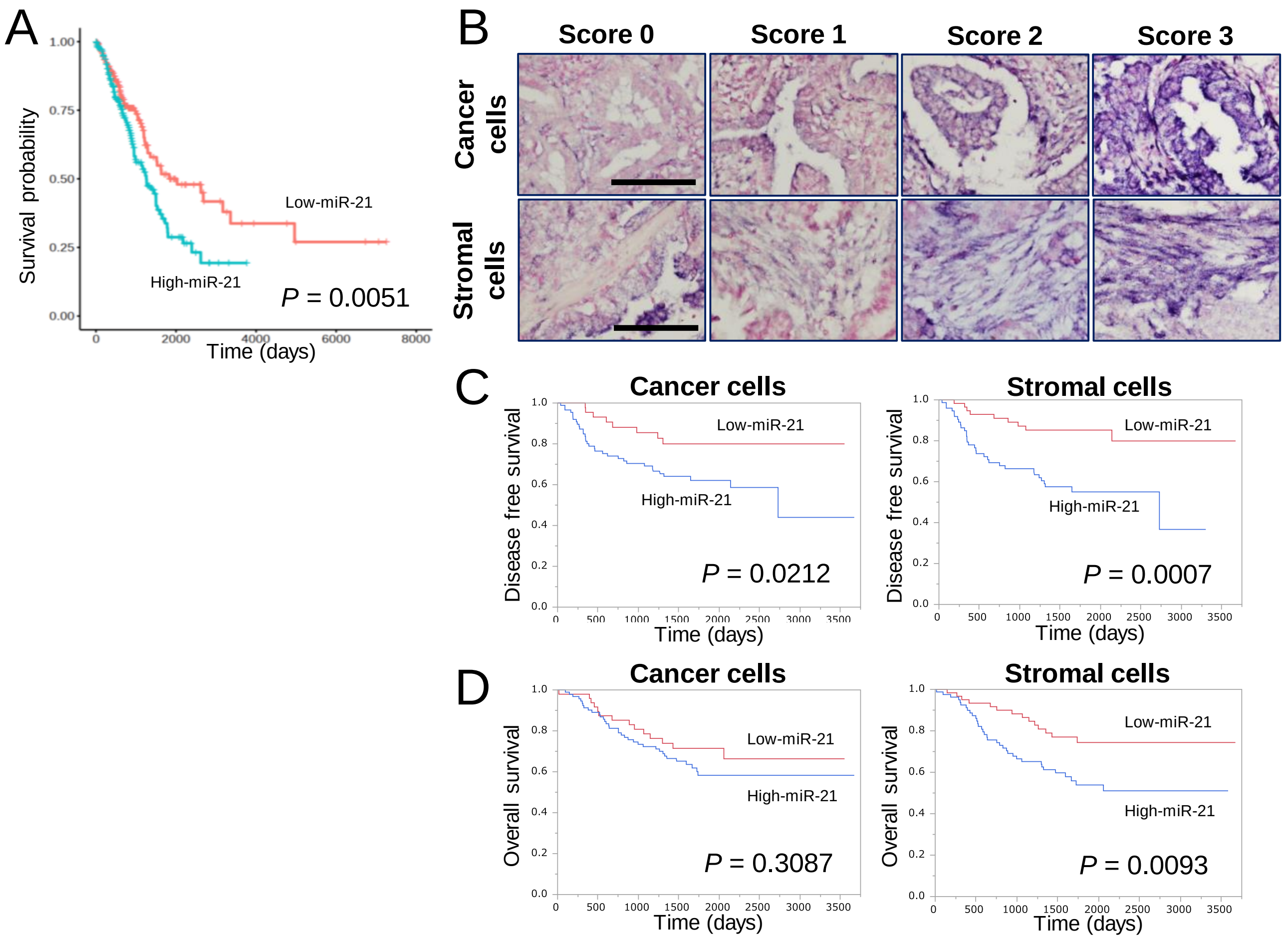

Supplementary Figure 1: Prognostic significances of microRNA-21 expression in lung adenocarcinoma.

(A) Overall Kaplan-Meier survival curves for 448 cases with The Cancer Genome Atlas (TCGA) data contrasting low versus high expression levels of microRNA-21 (miR-21). (B) Histoscores of *in situ* hybridization (ISH) for miR-21 for 144 cases with tissue microarray (TMA). Histoscores 2 and 3 are defined as the high expression group (bar, 100 μm). (C) Kaplan-Meier curves for disease-free survival contrasting low versus high expression levels of miR-21 in cancer cells and stromal cells, respectively. (D) Kaplan-Meier curves for overall survival contrasting low versus high expression levels of miR-21 in cancer cells and stromal cells, respectively. The *P* value is from the log-rank test.
