# Supplementary material for: Clinicopathological significance of microRNA‐21 in extracellular vesicles of pleural lavage fluid of lung adenocarcinoma and its functions inducing the mesothelial to mesenchymal transition
Source: Cancer Med. 2020 Feb 24;9(8):2879–90. doi: 10.1002/cam4.2928 (PMC7163097; doi:10.1002/cam4.2928)
Supplement: Supplementary file 2 [file CAM4-9-2879-s002.pdf]

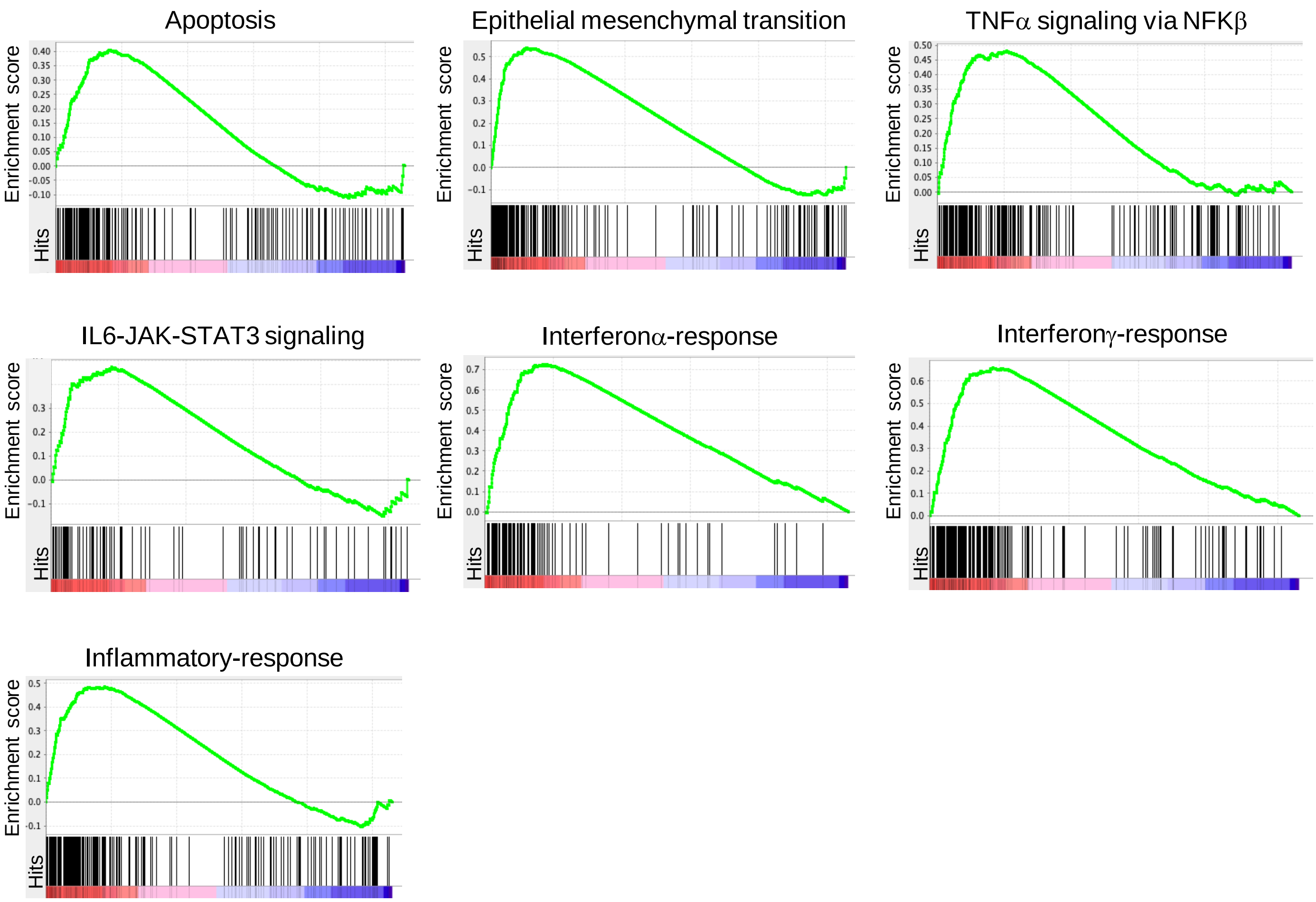

Supplementary Figure 2: Function of miR-21 in lung adenocarcinomas studied by GSEA using TCGA data.

GSEA demonstrates that upregulated gene sets in high-miR-21 expression cases are related to apoptosis, epithelial mesenchymal transition, TNFα signaling via NFKβ, IL6-JAK-STAT3 signaling, interferonα-response, interferonγ-response, and inflammatory-response (P<0.001).
