# Supplementary material for: Clinicopathological significance of microRNA‐21 in extracellular vesicles of pleural lavage fluid of lung adenocarcinoma and its functions inducing the mesothelial to mesenchymal transition
Source: Cancer Med. 2020 Feb 24;9(8):2879–90. doi: 10.1002/cam4.2928 (PMC7163097; doi:10.1002/cam4.2928)
Supplement: Supplementary file 3 [file CAM4-9-2879-s003.docx]

**Supplementary Table: Immunohistochemical markers of the mesothelial cells and fibroblasts**

|  | Calretinin | Desmin | αSMA |
| --- | --- | --- | --- |
| Mesothelial cells | + | + | - |
| Non-activated fibroblasts | - | - | - |
| CAFs (activated fibroblasts) | - | - | + |
| CAFs derived from mesothelial cells | + | + | + |

CAFs, cancer associated fibroblasts.
